# Postictal recovery of orientation in person, place and time relates to restoration of cortical activity after electroconvulsive therapy

**DOI:** 10.1192/j.eurpsy.2024.292

**Published:** 2024-08-27

**Authors:** S. Stuiver, J. Pottkämper, J. Verdijk, F. ten Doesschate, M. van Putten, J. Hofmeijer, J. van Waarde

**Affiliations:** ^1^Psychiatry, Rijnstate Hospital, Arnhem; ^2^University of Twente, Enschede; ^3^Neurology, Rijnstate Hospital, Arnhem; ^4^Clinical Neurophysiology, Medisch Spectrum Twente, Enschede, Netherlands

## Abstract

**Introduction:**

Most patients show temporary impairments in clinical orientation (i.e., orientation in person, place, and time) after electroconvulsive therapy (ECT)-induced seizures. It is unclear whether postictal reorientation is related to electroencephalography (EEG) restoration. This tentative relationship may shed light on mechanistic aspects of reorientation after ECT.

**Objectives:**

To study whether postictal EEG restoration after an ECT-induced seizure is related to recovery of clinical orientation in the cognitive domains person, place and time.

**Methods:**

We performed a longitudinal study in ECT patients and collected continuous postictal EEGs. Postictal EEG restoration was estimated by the evolution of the normalized alpha/delta ratio (ADR). Recovery of orientation in the cognitive domains of person, place, and time was assessed using the Reorientation Time (ROT) questionnaire. In each cognitive domain, a linear mixed model was fitted to investigate the relationship between ROT and postictal EEG restoration. In these models, other (ECT-)parameters including seizure duration, use of benzodiazepines and electrode placement were included.

**Results:**

In total, 272 ictal and postictal EEG recordings of 32 patients were included. In all domains, longer ROT was associated with slower postictal EEG recovery. Longer seizure duration and use of benzodiazepines were related to longer ROT in all domains. Increased total charge of the ECT-stimulus was associated with increased ROT in place and age was positively associated with ROT in time.

**Image:**

**Figure:**
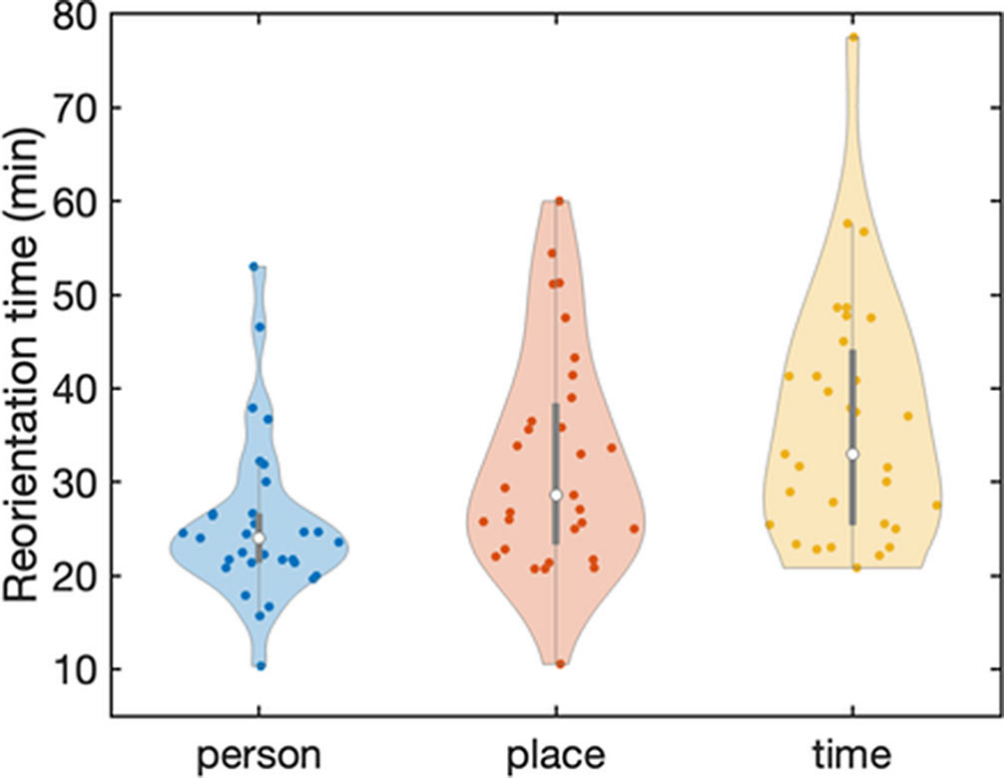

**Image 2:**

**Figure:**
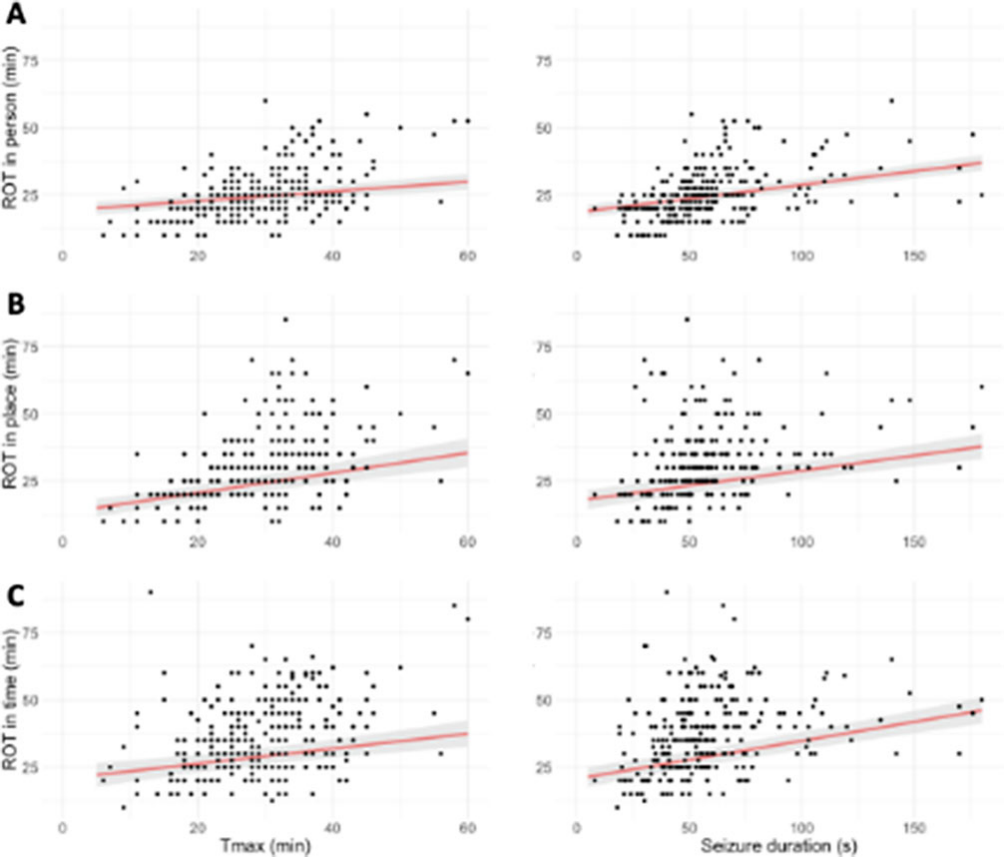

**Conclusions:**

We show a relationship between restoration of the postictal EEG and clinical reorientation in person, place and time after ECT-induced seizures. This indicates that clinical reorientation probably depends on gradual cortical synaptic recovery. Increased seizure duration and the use of benzodiazepines were also related to increased ROT values. Longer seizures and use of benzodiazepines may induce longer postictal synaptic depression.

**Disclosure of Interest:**

None Declared

